# Supplementary material for: G Protein-Coupled Receptor 37L1 Modulates Epigenetic Changes in Human Renal Proximal Tubule Cells
Source: Int J Mol Sci. 2022 Nov 21;23(22):14456. doi: 10.3390/ijms232214456 (PMC9698582; doi:10.3390/ijms232214456)
Supplement: Supplementary file 1 [file ijms-23-14456-s001.zip › ijms-1743098-supplementary.pdf]

Supplementary material

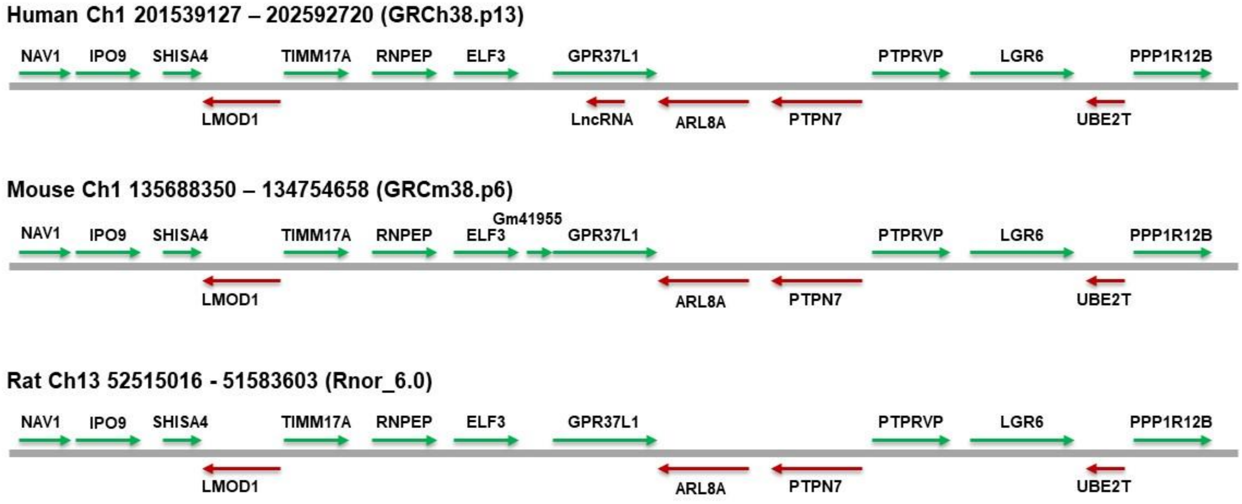

Figure S1. Genomic organization of the 1 MB hypertensive locus is highly similar among the rat, mouse, and human, except for LncRNA within the human GPR37L1 and a Gm41955 between ELF3 and GPR37L1 in the mouse.

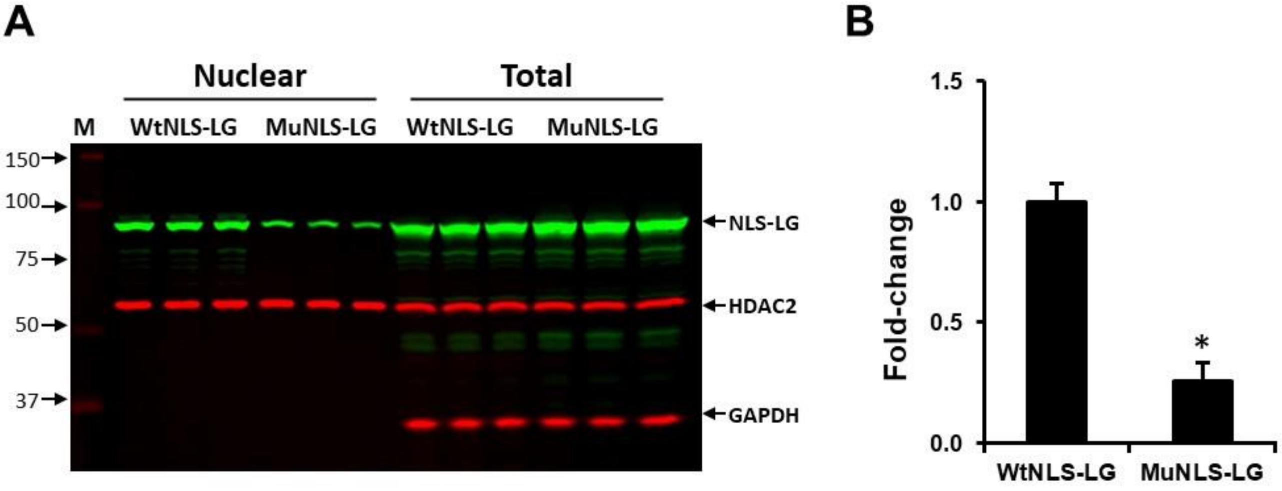

Figure S2. Basic amino-acid sequence RSKR in the predicted NLS is required for nuclear expression of GPR37L1. (**A**) hRPTCs were transfected with the wild-type NLS-LG (WtNLS-LG) or mutant NLS-LG (MuNLS-LG) plasmids. The expressions of WtNLS-LG and MuNLS-LG on nuclear or total protein preparations were determined by immunoblotting, using two-color infrared fluorescent protein detection. Wt or Mu- NLS-LG (green) were detected using the GFP antibodies. The blot was probed sequentially for HDAC2 (red), a marker for nuclear protein and GAPDH (red), a marker for cytoplasmic proteins. IRDye® 800CW-labeled donkey anti-rabbit IgG (926-32213, Licor, Lincoln, NE) was used for GPR37L1-GFP, and IRDye® 680RD-labeled donkey anti-mouse IgG (926-68022, Licor, Lincoln, NE) was used for HDAC2 and GAPDH. The molecular size markers are indicated on the left, and the Wt- or Mu- NLS-LG, HDAC2 and GAPDH proteins are indicated on the right. (**B**) Quantification of nuclear Wt- or Mu- NLS-LG expression was normalized to total Wt- or Mu- NLS-LG. n=3/group, *$P < 0.05$ vs WtNLS-LG, t-test.
